# Functional Genomics of Novel Secondary Metabolites from Diverse Cyanobacteria Using Untargeted Metabolomics

**DOI:** 10.3390/md11103617

**Published:** 2013-09-30

**Authors:** Richard Baran, Natalia N. Ivanova, Nick Jose, Ferran Garcia-Pichel, Nikos C. Kyrpides, Muriel Gugger, Trent R. Northen

**Affiliations:** 1 Life Sciences Division, Lawrence Berkeley National Laboratory, 1 Cyclotron Rd, MS977R0181A, Berkeley, CA 94720, USA; E-Mails: RBaran@lbl.gov (R.B.); njose40707@gmail.com (N.J.); 2 DOE Joint Genome Institute, Walnut Creek, CA 94598, USA; E-Mails: nnivanova@lbl.gov (N.N.I.); nckyrpides@lbl.gov (N.C.K.); 3 School of Life Sciences, Arizona State University, Tempe, AZ 85287, USA; E-Mail: ferran@asu.edu; 4 Institute Pasteur, Collection of Cyanobacteria, Paris Cedex 15 75724, France; E-Mail: muriel.gugger@pasteur.fr

**Keywords:** cyanobacteria, metabolomics, mass spectrometry, MS/MS, betaines, oligosaccharides

## Abstract

Mass spectrometry-based metabolomics has become a powerful tool for the detection of metabolites in complex biological systems and for the identification of novel metabolites. We previously identified a number of unexpected metabolites in the cyanobacterium *Synechococcus* sp. PCC 7002, such as histidine betaine, its derivatives and several unusual oligosaccharides. To test for the presence of these compounds and to assess the diversity of small polar metabolites in other cyanobacteria, we profiled cell extracts of nine strains representing much of the morphological and evolutionary diversification of this phylum. Spectral features in raw metabolite profiles obtained by normal phase liquid chromatography coupled to mass spectrometry (MS) were manually curated so that chemical formulae of metabolites could be assigned. For putative identification, retention times and MS/MS spectra were cross-referenced with those of standards or available sprectral library records. Overall, we detected 264 distinct metabolites. These included indeed different betaines, oligosaccharides as well as additional unidentified metabolites with chemical formulae not present in databases of metabolism. Some of these metabolites were detected only in a single strain, but some were present in more than one. Genomic interrogation of the strains revealed that generally, presence of a given metabolite corresponded well with the presence of its biosynthetic genes, if known. Our results show the potential of combining metabolite profiling and genomics for the identification of novel biosynthetic genes.

## 1. Introduction

Cyanobacteria are photoautotrophic bacteria capable of oxygenic photosynthesis. Members of the cyanobacteria phylum are important primary producers of organic matter in diverse ecosystems ranging from temperate terrestrial and marine to extreme environments [1,2,3,4]. Their broad ecological presence makes cyanobacteria important determinants of global geochemical cycles of carbon and nitrogen [5]. Cyanobacteria also commonly produce diverse secondary metabolites and bioactive compounds [6,7] and the ability of cyanobacteria to utilize solar energy and to fix carbon dioxide has drawn interest for biotechnological applications [8,9,10,11,12].

Improved understanding of cyanobacterial metabolites and their utilization have great potential for drug development. Unfortunately, functional characterization of microbial metabolism in general has lagged behind the pace of sequencing [13] and significant proportions of microbial genes have no assigned function as well as numerous biochemical activities are not assigned to any specific gene [14]. Microbial functional genomics has benefited from advances in comparative genomics [15], availability of bacterial expression [16] and mutant libraries [17], large scale phenotyping [18,19] and genome-scale metabolic modeling [20], all synergistically enabling advances in assigning functions to specific genes.

Mass spectrometry-based metabolomics is an established platform in microbial functional genomics [21,22]. Examples of successful assignments of gene function include incubations of complex mixtures of metabolites with purified proteins of unknown function to discovery enzymatic activities [23,24], and screening of libraries of bacterial mutants to identify genes of enzymes and transport proteins required for the utilization of specific metabolites [25]. Metabolite profiling often helps gene functional assignment but it more often points to novel metabolic capabilities. Uncharacterized biosynthetic capabilities are manifested by the frequent detection of novel metabolites or metabolites, which currently cannot be identified using mass spectrometry alone [26,27,28]. Additionally, utilization of uncharacterized metabolites from complex media or metabolite utilizations, which were not predicted based on available genome annotations, were also observed in bacteria [28,29].

Here we present untargeted metabolite profiling of cell extracts of nine additional cyanobacteria (Table 1) with available genome sequences [30,31,32] to test for the presence of unexpected metabolites previously detected in *Synechococcus sp.* PCC 7002 and to explore the diversity of small polar metabolites in this phylum. This exploratory study is intended to provide leads for downstream detailed structural and functional characterization of novel natural compounds.

**Table 1:** List of cyanobacteria used for metabolite profiling.

Taxonomic Subsection	Species	Strain	Abbreviation	Medium
1 (Chroococcales)	*Halothece* sp.	PCC 7418	7418	ASNIII/Tu4X
1 (Chroococcales)	*Synechococcus elongatus*	PCC 6301	6301	BG11
1 (Chroococcales)	*Synechococcus* sp.	PCC 7002	7002	ASNIII/BG11 + vit. B12
2 (Pleurocapsales)	*Chroococcidiopsis* sp.	PCC 6712	6712	ASNIII/BG11
2 (Pleurocapsales)	*Pleurocapsa* sp.	PCC 7327	7327	BG11
3 (Oscillatoriales)	*Geitlerinema* sp.	PCC 7407	7407	BG11
3 (Oscillatoriales)	*Leptolyngbya* sp.	PCC 7376	7376	ASNIII + vit. B12
3 (Oscillatoriales)	*Microcoleus vaginatus*	PCC 9802	9802	BG11
4 (Nostocales)	*Calothrix* sp.	PCC 7507	7507	BG11o
4 (Nostocales)	*Nostoc* sp.	PCC 7107	7107	BG11o

BG11 and ASNIII as in ref. [33]; BG11o, BG11 without nitrate; vit. B12, Vitamin B12 at 10 μg/L final concentration; Tu4X, Turks Island salts 4× concentration.

## 2. Results and Discussion

**Figure 1:**
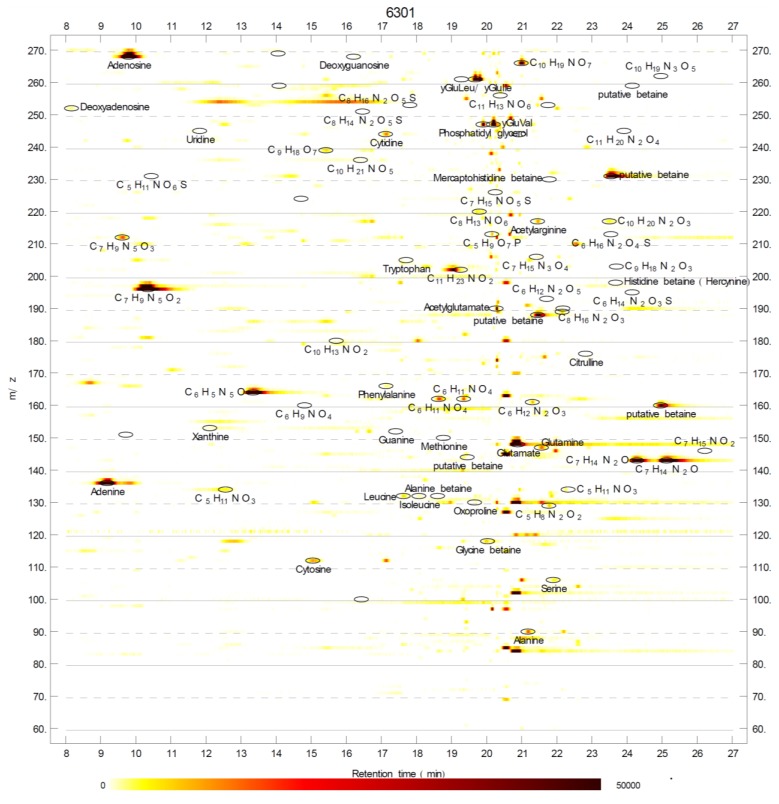
Three-dimensional visualization of a metabolite profile (from *Synechococcus elongatus* PCC 6301, partial). The X axis represents the retention time, the Y axis represents *m/z*, and the ion count intensity is color coded. Labels correspond to annotated metabolites (as found in Supplementary Table S1). Strong signals (>10,000 ion counts) without annotation labels correspond to redundant peaks (e.g., fragments or adducts) of annotated metabolites.

Analysis of raw metabolite profile data of selected cyanobacteria (Table 1) led to the curation of 264 metabolites across the ten analyzed cyanobacteria (Supplementary Table S1). The data analysis was performed combining the MathDAMP package [34] with iterative manual curation as described in the Experimental section. Annotations of metabolites were also visualized on density plots (Figure 1) to find any unannotated metabolites. Our aim was to annotate polar metabolites with at least one of its spectral features above 10,000 ion counts.

Tandem mass spectrometry (MS/MS) was performed on characteristic ions (primarily [M + H]^+^ in positive mode and [M − H]^−^ in negative mode) of metabolites following spectral feature annotation (Figure 1, Supplementary Table S1). Putative identifications of metabolites were based on our in-house database, built with identifications from our previous studies [26,29,30] and analysis of MS/MS spectra using public spectral libraries [35,36]. MS/MS spectra do not always provide sufficient information for full structural characterization of metabolites without corresponding true chemical standards, which limits the characterization power and overall degree of certainty of untargeted metabolomics [37]. Out of 264 metabolites annotated in this study, we could assign chemical formulae to 157 and putatively identify only 105 metabolites. This further underscores the relative ease of detecting metabolites which are not included in databases of metabolism or spectral libraries using untargeted metabolite profiling.

### 2.1. Betaines and Their Biosynthetic Genes

**Figure 2:**
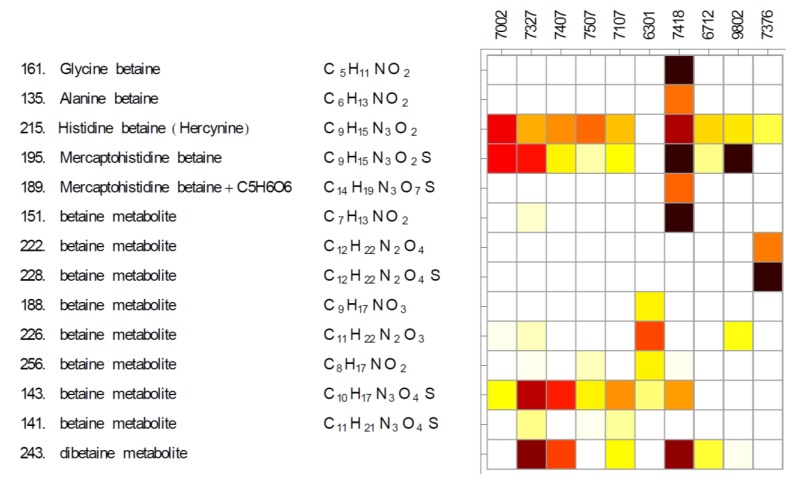
Diversity of betaines in analyzed cyanobacteria.

Betaines are neutral zwitterionic metabolites that contain a cationic group such as a quaternary ammonium, and a negatively charged carboxyl group, and whose known metabolic function are as compatible solutes (osmolites). We have previously identified histidine betaine (hercynine) and a thiol of histidine betaine (possibly ergothioneine) in *Synechococcus* sp. PCC 7002 based on MS/MS data [28]. The consistency of our MS/MS spectra with those of authentic standards was later confirmed [25,38]. The biosynthesis of hercynine and ergothioneine has been characterized in mycobacteria. Homologs of mycobacterial genes for some steps of the biosynthesis have been reported for cyanobacteria [39]. In fact the biosynthesis of hercynine and ergothioneine has been confirmed experimentally in five cyanobacterial strains [40]. In this study, we detected hercynine in nine out of the ten strains, and a thiol of hercynine in eight of these nine (Figure 2). The genomes of these nine cyanobacteria contain homologs of some mycobacterial genes corresponding to ergothioneine biosynthesis, while the genome of *Synechococcus elongatus* PCC 6301 lacks homologs of these genes (Supplementary Table S2). Glycine betaine was detected only in *Halothece* sp. PCC 7418 (Figure 2) what is consistent with the presence of the glycine methylation pathway [41]. Interestingly, this strain, which is a representative of the most halotolerant cyanobacteria known [42], contains the most diverse set of putative betaines of all strains tested.

Eleven additional metabolites were classified as betaines (Figure 2). Assignments to betaines were based on characteristic weak signals of their corresponding [M − H]^−^ ions in negative mode profile (MS1) mass spectra and the presence of trimethylamine-related fragments (*m/z* 60.0808, 59.0730, 58.0651) and trimethylamine neutral loss (59.0735) in positive mode MS/MS spectra (Supplementary Figure S1 and Table S1). Chemical formulae of some unidentified betaines correspond to a few candidate compounds (e.g., C_7_H_13_NO_2_ could correspond to crotonobetaine or proline betaine) but the analyses of MS/MS spectra were inconclusive. An interesting pair of betaines was detected exclusively in *Leptolyngbya* sp. PCC 7376. The chemical formulae of these two betaines differ by a single sulfur (C_11_H_22_N_2_O_4_, C_11_H_22_N_2_O_4_S)—an analogous pattern to hercynine and ergothioneine (Figure 2), pointing to a similar role. Accurate mass and isotopic profile of the betaine with metabolite number 243 is consistent with an oxidized form of ergothioneine (C_18_H_28_N_6_O_4_S_2_). This metabolite is also present in extracts of multiple strains in which the thiol of histidine betaine was detected (Figure 2). This coincidence and the mere presence of an oxidized form, would be consistent with an antioxidant role of ergothioneine [43].

### 2.2. Diversity of Glycosides and Oligosaccharides

**Figure 3:**
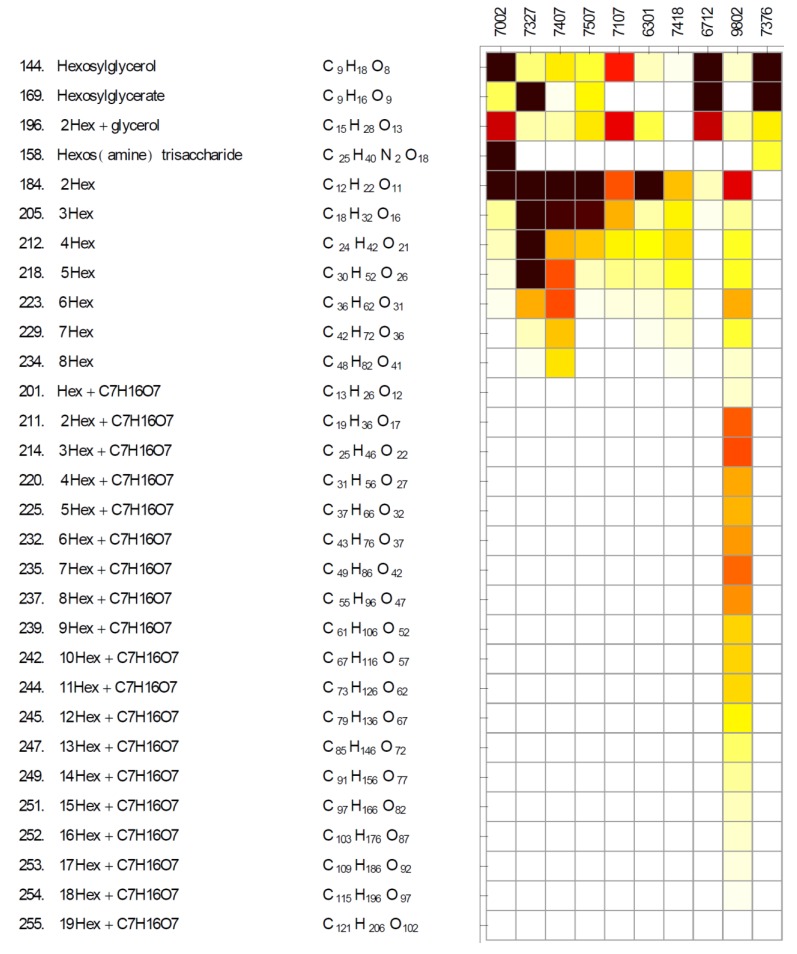
Diversity of glycosides and oligosaccharides in analyzed cyanobacteria.

Cyanobacteria are known to synthesize glycosides and oligosaccharides as compatible solutes [44], glycogen as a storage polysaccharide, and a variety of exopolysaccharides [45]. While glucosylglycerol and glucosylglycerate are common glycoside compatible solutes [44], other hexoses such as mannose and galactose may substitute for glucose [46]. Since we did not have authentic standards of these isomers to test if they have different chromatographic properties using our LC-MS method, we use combined names hexosylglycerol and hexosylglycerate for these glycerides (Figure 3). Homologs of glucosylglycerol-phosphate synthase and glucosylglycerol 3-phosphatase were identified by genomic annotation only in five of the strains analyzed (Supplementary Table S3), while hexosylglycerol was detected in all ten cyanobacteria (Figure 3, Supplementary Table S1). These findings suggest the presence of an alternative pathway for the biosynthesis of these glycerides. One simple possibility is transglycosylation reactions catalyzed by alpha-glucosidases, which have been shown to produce glucosylglycerol *in vitro* [47]. We detected hexosylglycerate in five of our strains (Figure 3, Supplementary Table S1). Homologs of glucosylglycerate biosynthetic genes were identified in four of these 5 cyanobacteria (Supplementary Table S4) showing overall good consistency between metabolite profiles and gene content while pointing to unusual hexosylglycerate biosynthesis in *Calothrix* sp. PCC 7507.

In addition to the detection of a hexose disaccharide, which could correspond to sucrose [44] or trehalose [31], a series of higher hexose oligomers was also detected (Figure 3). These could be storage glycogen-related maltooligosaccharides or structurally heterogeneous oligohexoses reported in different cyanobacteria [48,49]. Peak areas of ions of different metabolites cannot be used directly for absolute quantitative comparisons due to different ionization efficiencies of different metabolites [50]. However, results in Figure 3 clearly show different distribution of glycosides and oligosaccharides among the cyanobacteria studied here. Such comparisons may prove useful for identifying suitable starting points for engineering heterologous pathways or using cyanobacterial biomass as a feedstock for biotechnological applications.

We have previously identified an unusual trisaccharide of a hexose, *N*-acetylhexosamine and an oxidized version of *N*-acetylmuramic acid in *Synechococcus* sp. PCC 7002 [28]. This trisaccharide was detected as two distinct chromatographic peaks possibly caused by two anomers or mutarotation suggesting a reducing nature of this trisaccharide [28]. During this study, we detected this oligosaccharide in three additional cyanobacteria, also in two separate chromatographic peaks, yet at significantly lower signal intensities than in *Synechococcus* sp. PCC 7002 (Figure 3, Supplementary Table S1). Peak areas of the two peaks were combined and reported as a single metabolite number 158 (Supplementary Table S1). The role of this trisaccharide remains unclear; no other “decorated” hexosamine-based oligomers were identified in this study.

A range of unusual oligosaccharides was detected exclusively in *Microcoleus vaginatus* PCC 9802 (Figure 3). These oligosaccharides are condensation products of oligohexoses with C_7_H_16_O_7_. This chemical formula is consistent with a chemical formula of seven-carbon sugar alcohols. Release of polysaccharides is considered to play a key role in cyanobacterial gliding [51] and oligohexoses with C_7_H_16_O_7_ could be related to motility in *Microcoleus vaginatus*, a vertical migrant in desert soils [52]. Another possibility is that these glycans are related to exopolysaccharides which are known to play an important ecological role in binding soils to form biological soil crusts [53]. Certainly, the large comparative diversity of these compounds in *Microcoleus vaginatus* PCC 9802 suggests that they may play a differential physiological role related to adaptations to life in desert soils.

### 2.3. Gamma-Glutamyl Dipeptides and Gamma-Glutamyltransferase (ggt)

**Figure 4:**
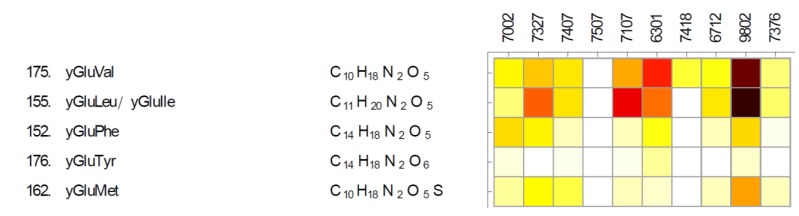
Distribution of gamma-glutamyl dipeptides in analyzed cyanobacteria.

A series of gamma-glutamyl dipeptides was among the unexpected findings in the profile of *Synechococcus* sp. PCC 7002 [28]. Gamma-glutamylation increases the solubility of non-polar amino acids [54] and may represent a strategy to prevent the loss of these amino acids via leakage through cell membranes [29]. Gamma-glutamyl dipeptides were detected in nine analyzed cyanobacteria and not detected in *Calothrix* sp. PCC 7507 (Figure 4). Homologs of gamma-glutamyltransferase gene (*ggt*) were identified in genomes of nine strains, interestingly; the strain missing the homolog is *Leptolyngbya* sp. PCC 7376 (Supplementary Table S5). *Calothrix sp.* PCC 7507 and two additional strains possess *ggt* sequences that are missing the catalytic threonine dyad shown to be important for autoprocessing in *Helicobacter pylori* *ggt* [55] (Supplementary Figure S2). This may indicate that *ggt* in *Calothrix* sp. PCC 7507 is not active or that it has some other (non-*ggt*) activity. *Pleurocapsa* sp. PCC 7327 has two additional *ggt* genes, at least one of them has perfectly conserved threonine dyad. And it is possible that both *Halothece* sp. PCC 7418, which has the same possibly inactive *ggt* as *Calothrix* sp. PCC 7507 and *Leptolyngbya* sp. PCC 7376, which has no *ggt* at all, have an alternative form of gamma-glutamyltransferase, which is not similar to the known *ggt*.

### 2.4. Other Metabolites

Similarly to the differences in the distribution of oligosaccharides, we also observed differences in the profiles of fatty acids, especially unsaturated ones (Supplementary Figure S3). A large proportion of detected metabolites remained unidentified and their chemical formulae are not present in databases of metabolism KEGG [56] or MetaCyc [57]. These metabolites represent a valuable resource for the characterization of novel metabolic capabilities. Having metabolite profile data of only ten cyanobacterial strains proved insufficient to link specific genes to individual metabolites as the number of candidate genes correlating with the presence or absence of specific metabolites was too large. Nevertheless, we believe that scaling up untargeted metabolite profiling to a larger number of strains would enable to both detect novel metabolites as well as to zoom in on a small number of potential corresponding biosynthetic genes.

## 3. Experimental Section

### 3.1. Strains and Culture Conditions

All strains are available at Pasteur Culture collection of Cyanobacteria [58]. Biomass of the nine PCC strains for chemical analyses was obtained by growing in 1.25 L of media suitable for the strain. The cultures were grown at 25 °C under continuous light provided by Osram Universal White fluorescent tubes (20 µmol quanta m^−2^ s^−1^) with agitation and constant bubbling of 1% CO_2_. Culture media are specified in Table 1. *Calothrix* sp. PCC 7507 and *Nostoc* sp. PCC 7107 are heterocystous cyanobacteria and these two strains were grown in nitrate free media to promote the development of heterocysts. All chemicals for growth media were purchased from Sigma.

### 3.2. Metabolite Extraction

Two milliliters of methanol were added to lyophilized biomass originating from approximately 2 mL of packed cell volume for each cyanobacterium (Table 1). In the case of Microcoleus vaginatus PCC 9802, 2 mL of methanol were added to cell pellet of approximately 1 mL of packed cell volume. The suspensions were sonicated for 15 min in sonic bath (VWR symphony) and then transferred to 2 mL microcentrifuge tubes and centrifuged for 10 min at 2348 × *g* using an Eppendorf 5424 centrifuge. Supernatants were transferred to 1.8 mL glass vials and dried down using Savant Speedvac Plus (SC210A). 100 μL of methanol were added to each vial and the vials were stored at −20 °C. Prior to analysis by LC-MS, the samples were filtered using 0.22 μm PVDF microcentrifuge filters (Millipore).

### 3.3. LC-MS Analysis

An Agilent capillary 1200 liquid chromatography system coupled to an Agilent 6520 ESI-Q-TOF mass spectrometer was used for LC-MS analysis. ZIC-HILIC column (3.5 μm, 100 Å, 150 × 1 mm) for normal phase liquid chromatography using analytical conditions as described previously [28]. Profile mode data (MS1) were acquired using a fast polarity switching mode. One LC-MS run was performed for a single sample of each strain. Fragmentation (MS/MS) spectra were acquired as two separate positive and negative polarity runs for each sample using data-dependent selection of precursor ions (Auto MS/MS) using collision energy of 10 V. Sample injection volume was 2 μL.

### 3.4. Data Analysis

Raw datasets from profile mode (MS1) analysis were exported to mzdata format using Agilent MassHunter Qualitative analysis software (B.05.00) and preprocessed by MathDAMP package [34] to unit mass resolution for comparative analysis. Differences among preprocessed datasets of the ten analyzed cyanobacteria were identified using MathDAMP by direct comparisons identifying outliers using quartile analysis [34]. Redundant spectral features potentially corresponding to a single metabolite (adducts, multimers, fragments) were grouped by correlation of their peak shape along the chromatographic dimension as described previously [29]. Resulting groups of spectral features were manually curated using Agilent MassHunter Qualitative Analysis software for chemical formula calculation. Putative identification of metabolites was based on our previous results [28,29] and analysis of MS/MS spectra against spectral libraries Metlin [35] and MassBank [36].

### 3.5. Genome Analysis

Candidate genes and synthesis pathways were identified using the data and tools in Integrated Microbial Genomes database (IMG) [59]. GenBank accession numbers of 10 cyanobacterial genomes are as follows: CP003943 (*Calothrix* sp. PCC 7507), (*Chroococcidiopsis* sp. PCC 6712), CP003591 (*Geitlerinema* sp. PCC 7407), CP003945 (*Halothece* sp. PCC 7418), CP003946 (*Leptolyngbya* sp. PCC 7376), CP003548 (*Nostoc* sp. PCC 7107), CP003590 (*Pleurocapsa* sp. PCC 7327), AP008231 (*Synechococcus elongatus* PCC 6301), CP000951 (*Synechococcus* sp. PCC 7002). Putative orthologs of experimentally characterized proteins in cyanobacterial genomes have been identified as bi-directional best BLASTp [60] hits using e-value cutoffs of 1.0 × 10^−5^. Experimentally characterized proteins included in the analysis were ergothioneine biosynthesis proteins EgtB-E from *Mycobacterium smegmatis* MC2 155 [39], ovothiol biosynthesis protein OvoA from *Erwinia tasmaniensis* [61], GSMT and SDMT from *Ectothiorhodospira halochloris* [41], ggpPS from *Synechocystis* sp. PCC 6803 [62] and ggt from *H. pylori* [55]. Additional candidate genes in ergothioneine biosynthesis and gamma-glutamyltranspeptidase family proteins were identified by the matches to the corresponding TIGRfam models [63] and COG position-specific scoring matrices obtained from the CDD database [64].

## 4. Conclusions

In this study, we have shown that it is possible to correlate the presence of metabolites with known biosynthetic genes to the gene content of ten analyzed cyanobacteria. Additionally, we detected a series of novel betaines in some cyanobacteria and unusual oligohexoses with a degree of polymerization up to 19 with a single C_7_H_16_O_7_ moiety in *Microcoleus vaginatus* PCC 9802. The scale-up of such comparative metabolite profiling may enable the linking of genes of unknown function to the biosynthesis of novel natural compounds.

## Supplementary Files

Supplementary File 1 Supplementary (XLS, 128 KB)

Supplementary File 2 Supplementary Information (PDF, 568 KB)

Supplementary File 3 Supplementary msms data (ZIP, 1417 KB)
