# Supplementary material for: Functional Genomics of Novel Secondary Metabolites from Diverse Cyanobacteria Using Untargeted Metabolomics
Source: Mar Drugs. 2013 Sep 30;11(10):3617–31. doi: 10.3390/md11103617 (PMC3826126; doi:10.3390/md11103617)
Supplement: Supplementary File 2 — Supplementary Information (PDF, 568 KB) [file marinedrugs-11-03617-s002.pdf]

Supplementary Information

Table of Contents

**Table S1**	Separate xls file
**Table S2**	
**Table S3**	
**Table S4**	
**Table S5**	
**Figure S1**	
**Figure S2**	
**Figure S3**	
**MS/MS data**	Separate zip file

Table S2. Predicted ergothioneine biosynthesis genes across the genomes of analyzed cyanobacteria. No hercinylcysteine sulfoxide synthase (glutamine amidotransferase family protein) is found in the operon or elsewhere in the genome of *Synechococcus* PCC 7002. No ortholog of EgtE is present in any of the genomes; lyase reaction is probably catalyzed by another aminotransferase 5 family enzyme, although none is found in an operon with other ergothioneine synthesis genes. [1]—40% identity to *N*-terminal domain of *E. tasmaniensis* OvoA (ETA_0030); [2]—no similarity to *C*-terminal domain of *E. tasmaniensis* OvoA (ETA_0030), 47% identity to *M. smegmatis* EgtD (MSMEG_6247).

Genome	IMG Gene ID	Locus Tag	Gene Product Name	Function
Calothrix sp. PCC 7507	2505803781	Cal7507_5459	Sulphatase-modifying factor protein	predicted 5-histidylcysteine sulfoxide synthase [1]
Calothrix sp. PCC 7507	2505803782	Cal7507_5460	methyltransferase	predicted 5-histidylcysteine sulfoxide methyltransferase [2]
Chroococcidiopsis sp. PCC 6712	2505784276	Chr6712_0536	methyltransferase	hercynine synthase
Chroococcidiopsis sp. PCC 6712	2505785000	Chr6712_1252	Conserved hypothetical protein CHP03440	hercynyl-gamma-glutamylcysteine sulfoxide synthase
Chroococcidiopsis sp. PCC 6712	2505785001	Chr6712_1253	Conserved hypothetical protein CHP03442	hercynylcysteine sulfoxide synthase
Geitlerinema sp. PCC 7407	2503605984	GEI7407_0022	methyltransferase	hercynine synthase
Geitlerinema sp. PCC 7407	2503605985	GEI7407_0023	protein of unknown function DUF323	hercynyl-gamma-glutamylcysteine sulfoxide synthase
Geitlerinema sp. PCC 7407	2503605986	GEI7407_0024	glutamine amidotransferase class-II	hercynylcysteine sulfoxide synthase
Halothece sp. PCC 7418	2503638506	PCC7418_3752	methyltransferase	hercynine synthase
Halothece sp. PCC 7418	2503636330	PCC7418_1605	protein of unknown function DUF323	hercynyl-gamma-glutamylcysteine sulfoxide synthase
Halothece sp. PCC 7418	2503636329	PCC7418_1604	glutamine amidotransferase class-II	hercynylcysteine sulfoxide synthase
Leptolyngbya sp. PCC 7376	2503885948	Lepto7376_0353	methyltransferase	hercynine synthase

Table S2. *Cont.*

Leptolyngbya sp. PCC 7376	2503886757	Lepto7376_1155	Conserved hypothetical protein CHP03440	hercynyl-gamma-glutamylcysteine sulfoxide synthase
Leptolyngbya sp. PCC 7376	2503886756	Lepto7376_1154	Conserved hypothetical protein CHP03442	hercynylcysteine sulfoxide synthase
Microcoleus vaginatus PCC 9802	2505165825	Mvag_PCC9802_DRAFT2_00002240	probable methyltransferase	hercynine synthase
Microcoleus vaginatus PCC 9802	2505165824	Mvag_PCC9802_DRAFT2_00002230	TIGR03440 family protein	hercynyl-gamma-glutamylcysteine sulfoxide synthase
Microcoleus vaginatus PCC 9802	2505165823	Mvag_PCC9802_DRAFT2_00002220	TIGR03442 family protein	hercynylcysteine sulfoxide synthase
Nostoc sp. PCC 7107	2503739909	Nos7107_1277	methyltransferase	hercynine synthase
Nostoc sp. PCC 7107	2503739910	Nos7107_1278	Conserved hypothetical protein CHP03440	hercynyl-gamma-glutamylcysteine sulfoxide synthase
Nostoc sp. PCC 7107	2503739911	Nos7107_1279	Conserved hypothetical protein CHP03442	hercynylcysteine sulfoxide synthase
Pleurocapsa sp. PCC 7327	2509575893	Ple7327_4010	probable methyltransferase	hercynine synthase
Pleurocapsa sp. PCC 7327	2509576015	Ple7327_4133	TIGR03440 family protein	hercynyl-gamma-glutamylcysteine sulfoxide synthase
Pleurocapsa sp. PCC 7327	2509576019	Ple7327_4137	TIGR03442 family protein	hercynylcysteine sulfoxide synthase
Synechococcus sp. PCC 7002	641610000	SYNPCC7002_G0154	hypothetical protein	hercynine synthase
Synechococcus sp. PCC 7002	641610001	SYNPCC7002_G0155	conserved hypothetical protein, domain of unknown function (DUF323)	hercynyl-gamma-glutamylcysteine sulfoxide synthase

Table S3. Predicted glucosylglycerol biosynthesis genes across the ten analyzed cyanobacteria.

Genome	IMG Gene ID	Locus Tag	Gene Product Name	Function
Chroococcidiopsis sp. PCC 6712	2505786553	Chr6712_2792	glucosylglycerol-phosphate synthase	glucosylglycerol-phosphate synthase
Chroococcidiopsis sp. PCC 6712	2505787835	Chr6712_4056	glucosylglycerol 3-phosphatase	glucosylglycerol 3-phosphatase
Halothece sp. PCC 7418	2503634797	PCC7418_0098	glucosylglycerol-phosphate synthase	glucosylglycerol-phosphate synthase
Halothece sp. PCC 7418	2503637004	PCC7418_2274	glucosylglycerol 3-phosphatase	glucosylglycerol 3-phosphatase
Leptolyngbya sp. PCC 7376	2503886392	Lepto7376_0792	glucosylglycerol-phosphate synthase	glucosylglycerol-phosphate synthase
Leptolyngbya sp. PCC 7376	2503886396	Lepto7376_0796	glucosylglycerol 3-phosphatase	glucosylglycerol 3-phosphatase
Synechococcus sp. PCC 7002	641612860	SYNPCC7002_A2851	glucosylglycerol-phosphate synthase	glucosylglycerol-phosphate synthase
Synechococcus sp. PCC 7002	641612851	SYNPCC7002_A2841	glucosylglycerol 3-phosphatase	glucosylglycerol 3-phosphatase
Geitlerinema sp. PCC 7407	2503608226	GEI7407_2224	glucosylglycerol-phosphate synthase	glucosylglycerol-phosphate synthase
Geitlerinema sp. PCC 7407	2503608225	GEI7407_2223	sucrose-6F-phosphate phosphohydrolase	predicted glucosylglycerol 3-phosphatase

Table S4. Predicted glucosylglycerate biosynthesis genes across the ten analyzed cyanobacteria.

Genome	IMG Gene ID	Locus Tag	Gene Product Name	Function
Chroococcidiopsis sp. PCC 6712	2505788254	Chr6712_4470	cell wall biogenesis glycosyltransferase	glucosyl-3-phosphoglycerate synthase
Chroococcidiopsis sp. PCC 6712	2505788418	Chr6712_4632	mannosyl-3-phosphoglycerate phosphatase family	glucosyl-3-phosphoglycerate phosphatase
Leptolyngbya sp. PCC 7376	2503885969	Lepto7376_0374	hypothetical protein	glucosyl-3-phosphoglycerate synthase
Leptolyngbya sp. PCC 7376	2503885971	Lepto7376_0376	mannosyl-3-phosphoglycerate phosphatase family	glucosyl-3-phosphoglycerate phosphatase
Pleurocapsa sp. PCC 7327	2509573393	Ple7327_1510	hypothetical protein	glucosyl-3-phosphoglycerate synthase
Pleurocapsa sp. PCC 7327	2509576101	Ple7327_4219	HAD-superfamily hydrolase, subfamily IIB	glucosyl-3-phosphoglycerate phosphatase
Synechococcus sp. PCC 7002	641612031	SYNPCC7002_A2021	hypothetical protein	glucosyl-3-phosphoglycerate synthase
Synechococcus sp. PCC 7002	641612033	SYNPCC7002_A2023	putative hydrolase, HAD superfamily protein	glucosyl-3-phosphoglycerate phosphatase

Table S5. Predicted gamma-glutamyltransferases and gamma-glutamyltransferase-like genes across the ten analyzed cyanobacteria.

Genome	IMG Gene ID	Locus Tag	Product Name	Protein family assignment
Calothrix sp. PCC 7507	2505801690	Cal7507_3414	Gamma-glutamyltransferase	COG0405, pfam01019
Chroococcidiopsis sp. PCC 6712	2505787521	Chr6712_3744	gamma-glutamyltransferase	COG0405, pfam01019
Geitlerinema sp. PCC 7407	2503609724	GEI7407_3705	gamma-glutamyltransferase	COG0405, pfam01019
Halothece sp. PCC 7418	2503637006	PCC7418_2276	Gamma-glutamyltransferase	COG0405, pfam01019
Microcoleus vaginatus PCC 9802	2505170787	Mvag_PCC9802_DRAFT2_00051890	gamma-glutamyltranspeptidase	COG0405, pfam01019
Nostoc sp. PCC 7107	2503740642	Nos7107_2002	gamma-glutamyltransferase	COG0405, pfam01019
Pleurocapsa sp. PCC 7327	2509572868	Ple7327_0985	gamma-glutamyltranspeptidase	COG0405, pfam01019
Pleurocapsa sp. PCC 7327	2509573859	Ple7327_1976	Gamma-glutamyltransferase	COG0405, pfam01019
Pleurocapsa sp. PCC 7327	2509574431	Ple7327_2548	gamma-glutamyltranspeptidase	COG0405, pfam01019
Synechococcus elongatus PCC 6301	637616990	syc1325_c	gamma-glutamyltranspeptidase	COG0405, pfam01019
Synechococcus sp. PCC 7002	641609980	SYNPCC7002_G0134	Gamma-glutamyltranspeptidase precursor	COG0405, pfam01019

Figure S1. Positive mode MS/MS spectra of putative betaines listed in Figure 2 of the main text (collision energy 10 V).

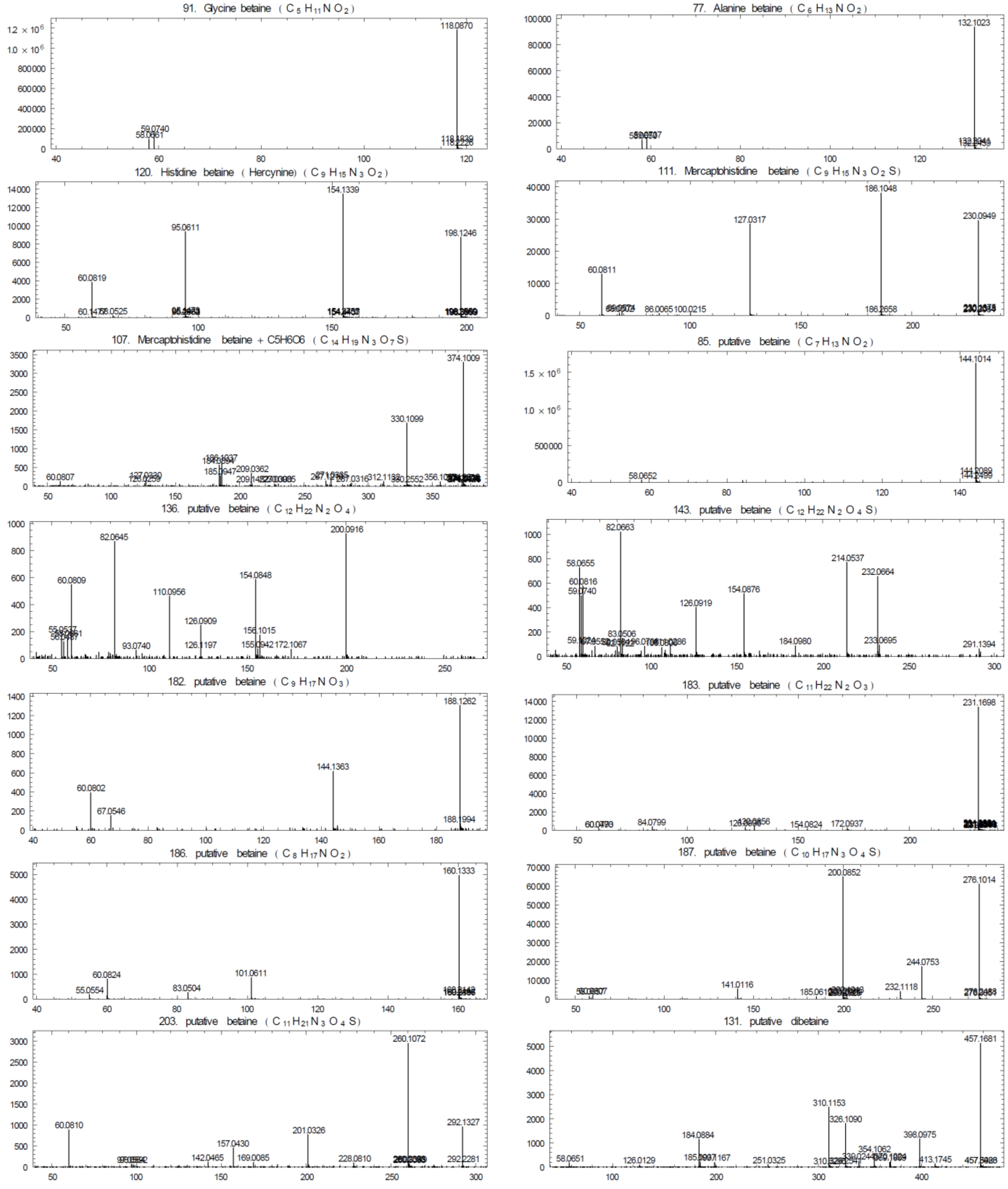

Figure S2. Partial multiple sequence alignment of gamma-glutamyltransferase-like genes from 10 cyanobacterial genomes with experimentally characterized reference sequences (*H. pylori* ggt and *B. anthracis* CapD). The peptide corresponding to the catalytic threonine dyad is highlighted in red. Alignment has been performed using ClustalX [1] and visualized using Jalview [2].

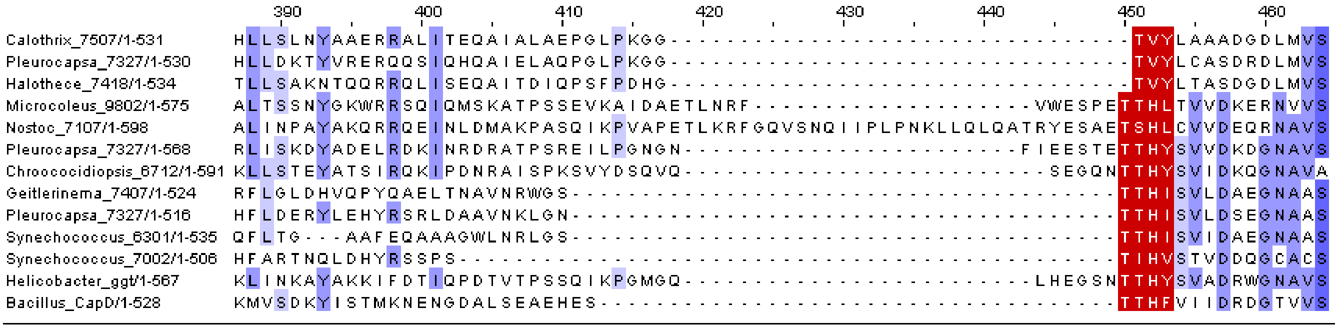

Figure S3. Diversity of fatty acids across the ten analyzed cyanobacteria.

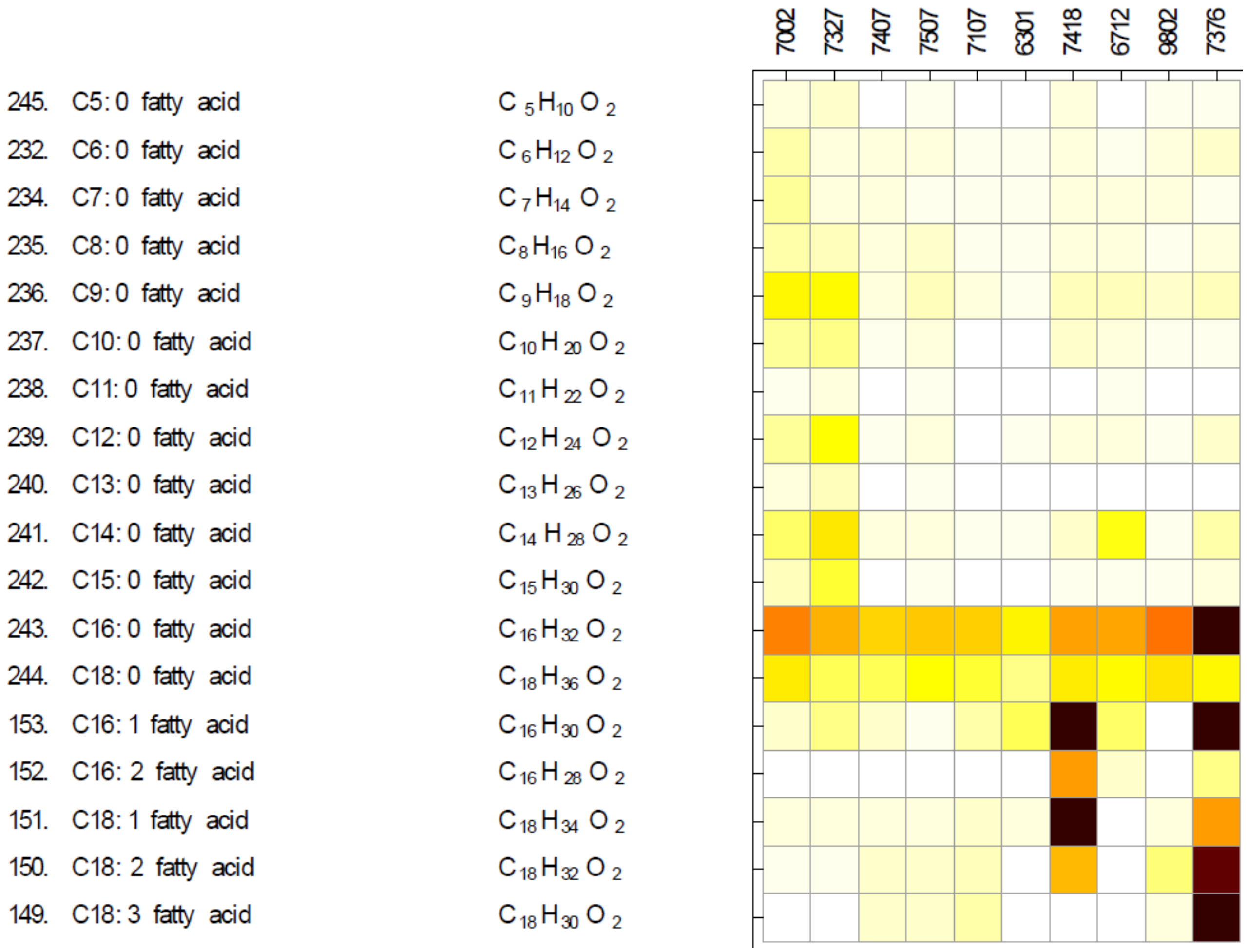

References

1. Larkin, M.A.; Blackshields, G.; Brown, N.P.; Chenna, R.; McGettigan, P.A.; McWilliam, H.; Valentin, F.; Wallace, I.M.; Wilm, A.; Lopez, R.; *et al.* Clustal W and Clustal X version 2.0. *Bioinformatics* **2007**, *23*, 2947–2948.
2. Waterhouse, A.M.; Procter, J.B.; Martin, D.M.; Clamp, M.; Barton, G.J. Jalview Version 2—a multiple sequence alignment editor and analysis workbench. *Bioinformatics* **2009**, *25*, 1189–1191.

© 2013 by the authors; licensee MDPI, Basel, Switzerland. This article is an open access article distributed under the terms and conditions of the Creative Commons Attribution license (http://creativecommons.org/licenses/by/3.0/).
